# Episodic transient deformation revealed by the analysis of multiple GNSS networks in the Noto Peninsula, central Japan

**DOI:** 10.1038/s41598-023-35459-z

**Published:** 2023-06-12

**Authors:** Takuya Nishimura, Yoshihiro Hiramatsu, Yusaku Ohta

**Affiliations:** 1 Disaster Prevention Research Institute, Kyoto University, Gokasho, Uji, Kyoto 611-0011 Japan; 2 Institute of Science and Engineering, Kanazawa University, Kakuma-cho, Kanazawa, Ishikawa 920-1192 Japan; 3 Graduate School of Science, Tohoku University, Aza-Aoba 6-6, Aramaki, Aoba-ku, Sendai, Miyagi 980-8578 Japan

**Keywords:** Geodynamics, Seismology, Tectonics

## Abstract

Since November 30, 2020, an intense seismic swarm and transient deformation have been continuously observed in the Noto Peninsula, central Japan, which is a non-volcanic/geothermal area far from major plate boundaries. We modeled transient deformation based on a combined analysis of multiple Global Navigation Satellite System (GNSS) observation networks, including one operated by a private sector company (SoftBank Corp.), relocated earthquake hypocenters, and tectonic settings. Our analysis showed a total displacement pattern over 2 years shows horizontal inflation and uplift of up to ~ 70 mm around the source of the earthquake swarm. In the first 3 months, the opening of the shallow-dipping tensile crack had an estimated volumetric increase of ~ 1.4 × 10^7^ m^3^ at a depth of ~ 16 km. Over the next 15 months, the observed deformation was well reproduced by shear-tensile sources, which represent an aseismic reverse-type slip and the opening of a southeast-dipping fault zone at a depth of 14–16 km. We suggest that the upwelling fluid spread at a depth of ~ 16 km through an existing shallow-dipping permeable fault zone and then diffused into the fault zone, triggering a long-lasting sub-meter aseismic slip below the seismogenic depth. The aseismic slip further triggered intense earthquake swarms at the updip.

## Introduction

An earthquake swarm is a sequence of earthquakes that lasts from several hours to several years without a distinct mainshock^1^. Earthquake swarms are often interpreted as phenomena related to external transient stress perturbations, including slow slip events^2^ and magma intrusion^3^, or weakening of fault strength due to an increase in pore fluid pressure^4^. Many earthquake swarms have been observed in areas with active volcanism, hydrothermal systems, transform faults, and subduction margins^5^.

**Figure 1:**
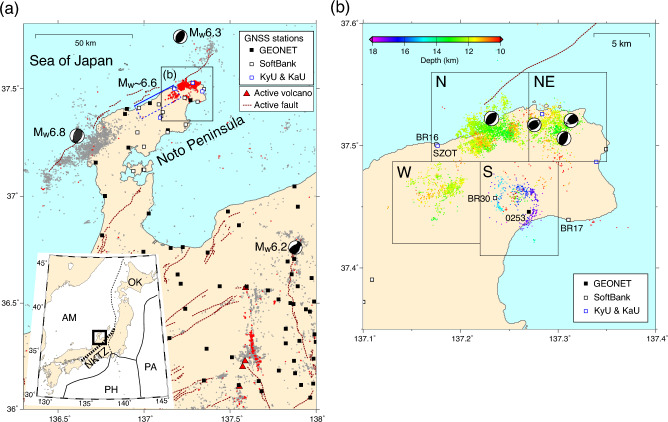
Epicenters of crustal earthquakes and GNSS stations around the Noto Peninsula, central Japan. Brown dotted lines and red triangles indicate surface traces of major active faults^7,13^ and active volcanoes^14^, respectively. Black solid, black open, and red open squares indicate GNSS stations (see legend). (**a**) Earthquake epicenters with M ≥ 2 and depth ≤ 20 km during 1998–2022 using the JMA catalog. Gray and red dots represent the epicenters before December 2020 and after November 2020, respectively. Focal mechanisms of M_w_ ≥ 6 crustal earthquakes since 1980 are indicated using the Global Centroid Moment Tensor catalog^15,16^. The source area of the 1729 M_w_ ~ 6.6 earthquake^12^ is indicated. (Inset) Tectonic map of Japan. AM, OK, PA, and PH indicate the Amurian, Okhotsk, Pacific, and Philippine Sea plates, respectively. NKTZ denotes the Niigata-Kobe Tectonic Zone^17^. (**b**) Relocated epicenters of M ≥ 1.2 earthquakes from November 2020 to November 2022. Four-digit codes show the ID of selected GNSS stations. Focal mechanisms of M ≥ 4.5 earthquakes during the swarm are indicated using the JMA catalog.

An intense crustal seismic swarm started at the end of November 2020 at the tip of the Noto Peninsula in central Japan, where microseismicity was rather low before the swarm (Fig. 1). By the end of February 2023, the total number of earthquakes with magnitude ≥ 1 [Japan Meteorological Agency (JMA) scale] had exceeded 14,000. The weekly number of M ≥ 1 earthquakes continued to exceed 90 for 80 weeks. The largest M5.4 earthquake occurred on July 19, 2022. Although the swarm region experienced active volcanism related to back-arc rifting that ceased 15 Ma, neither Holocene active volcanoes nor Quaternary volcanoes are observed within a radius of 50 km from the swarm region^6^. A major tectonic structure in the study area is an offshore active fault system along the northern coast of the Noto Peninsula^7^ (Fig. 1). Most active faults in the back-arc region of northeastern Japan, including the Noto Peninsula, were formed as normal faults related to the opening of the back-arc before 14 Ma, and they have been reactivated as reverse faults under the compressional stress regime^8,9^ in terms of inversion tectonics. Several large crustal earthquakes, including the 2007 M_w_6.8 Noto Hanto earthquake^10,11^ and the 1729 M_w_ ~ 6.6 earthquake^12^, occurred around the Noto Peninsula. The location and reverse-fault-type mechanism of these earthquakes suggest that they were caused by the offshore reverse fault system along the northern coast (Fig. 1a). Local residents have requested information on how long the swarm activity will continue and whether the present swarm activity is a precursor of a future large earthquake. To answer these questions, the mechanisms underlying ongoing seismic swarms must be better understood.

A key factor in understanding this mechanism is the geodetic observation of crustal deformation. The deformation of the Japanese Islands is monitored by a continuous Global Navigation Satellite System (cGNSS) network named the GNSS Earth Observation Network System (GEONET) by the Geospatial Authority of Japan (GSI)^18^. A GEONET station near the swarm area has shown significant uplift since the end of 2020. However, the existing GEONET stations, which have a density of one station per about 20 × 20 square kilometers, are not sufficient to adequately capture the detailed spatial patterns of shallow tectonic events in the crust. Therefore, after recognizing the earthquake swarm in the summer of 2021, our group and the GSI installed eight temporal cGNSS stations. However, the deformation that occurred at the beginning of the swarm could not be clarified by these temporal stations. Recently, private companies have constructed their own cGNSS networks^19,20^ owing to the rapid growth in GNSS applications for real-time high-precision positioning, including autonomous vehicles. SoftBank Corp. (SoftBank) operates a cGNSS network consisting of approximately 3300 continuous stations all over Japan. Ohta and Ohzono^21^ demonstrated that with appropriate data quality control, the GNSS data acquired through the SoftBank network are useful for monitoring, although the long-term stability (> 1 year) of these data has not been examined. The use of SoftBank GNSS data is expected to reveal transient deformation associated with an ongoing seismic swarm with an average spacing of less than 10 km.

In this study, we analyzed GNSS data acquired from multiple networks operated by SoftBank, GSI, Kyoto University, and Kanazawa University to clarify the spatiotemporal evolution of the transient deformation in the Noto Peninsula. We also relocated the earthquake hypocenters of the seismic swarm to compare them with the deformation sources. Finally, we estimated the deformation sources and proposed a mechanism for the intense seismic swarm, based on the observed deformation, seismicity, and tectonic background.

## Observed seismicity

The seismic swarm in the Noto Peninsula exhibited a spatial distribution that revealed four distinct clusters, which we refer to as Clusters S, W, N, and NE (Fig. 1b). This article provides a brief overview of the temporal evolution of the earthquake swarm. The seismic activity began in Cluster S in 2018. Burst-type activity occurred in the summer of 2018, and the depth of the earthquakes gradually became shallower (Supplementary Fig. S1). On November 30, 2020, earthquakes at depths of ≥ 15 km suddenly reactivated. The activity of Cluster S after reactivation was characterized by intermittent and burst-type swarms in time, whereas that of the other clusters showed a gradual increase for the first several months and kept a steady seismicity rate over a year (Supplementary Fig. S2). Seismicity was activated in the clockwise order of Clusters W, N, and NE (Supplementary Figs. S2 and S3). Precisely relocated hypocenters showed that the earthquakes formed southeast dipping-bands in each cluster and an overall southeast-dipping listric fault zone, as shown along cross sections D–D′ in Supplementary Fig. S3. However, the fine-scale structure showed that the earthquakes did not align along a single plane but rather formed multiple sub-parallel and conjugate planes. Earthquakes in Clusters W, N, and NE occurred mostly at depths of 10–14 km (Fig. 1b and Supplementary Fig. S3).

The focal mechanism of most earthquakes within the swarm was a reverse-fault type with the P-axis in the northwest-southeast direction, according to the F-net focal mechanism of the National Research Institute for Earth Science and Disaster Resilience (NIED)^22^. This fault type is consistent with the type of active faults and stress states estimated from the focal mechanisms of pre-swarm earthquakes^23,24^ (Fig. 1). More detailed analyses of the seismicity can be found in other studies^25,26^.

## Observed deformation and source model

**Figure 2:**
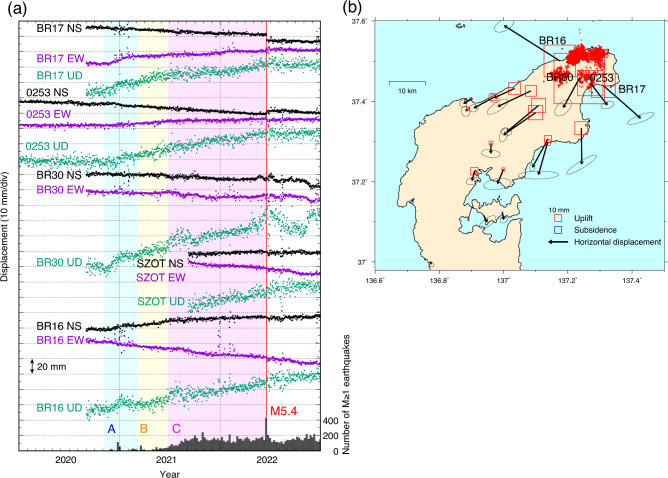
Transient deformation at selected GNSS stations. The secular motion before the transient deformation and some local motions are corrected (see text). (**a**) GNSS time-series at selected stations. The weekly number of M ≥ 1 earthquakes is plotted at the bottom. (**b**) Displacement from November 1–10, 2020, to December 22–31, 2022. Error ellipsoids represent 68% confidence intervals. Red dots show relocated epicenters of M ≥ 1.2 earthquakes from November 2020 to November 2022.

A time series of the corrected GNSS coordinates (Fig. 2a and Supplementary Fig. S4) clearly demonstrated that transient deformation started at the end of November 2020, which synchronized with the reactivation of deep earthquakes in Cluster S. The deformation rate was rapid at the beginning and decelerated in the first three months, which is clearly recognized in the EW component of BR17 and the UD component of BR30 from November 2020 to February 2021. The deformation continued at a nearly constant rate with some fluctuations until the M5.4 earthquake on June 19, 2022. Large coseismic offsets may contaminate monument instability due to strong ground shaking (Supplementary Fig. S5a). After the M5.4 earthquake, the deformation rates further decelerated at most stations, although significant deformation has continued at some stations as of February 2023 (e.g., BR16 and BR30) (Supplementary Fig. S5b). The total displacement from November 2020 to December 2022 showed a radial horizontal pattern and a broad uplift within ~ 40 km of the swarm area (Fig. 2b). The maximum uplift was approximately 70 mm at BR30.

Despite dense GNSS networks, the observed displacement was insufficient to resolve a source model due to limited station coverage in the peninsula and source depth. A point inflation source, a shallow-dipping tensile crack, and reverse slip of a southeast-dipping fault could roughly reproduce the observed displacement, whereas the first two sources and the last source can produce a significantly different displacement pattern in offshore regions (Supplementary Figs. S6 and S7; Supplementary Table S1). Therefore, we inferred the type and various parameters of the deformation sources based on seismic activity and tectonic settings. We tested trial-and-error modeling, assuming the above three types, and a combination of shear and opening motion on the same fault plane (e.g., shear tensile fault) based on natural^27^ and anthropogenic^28^ earthquake swarm sources. We focused on the transient deformation before the M5.4 earthquake and divided it into Periods A, B, and C based on the deformation pattern and seismicity as specified below. A key component of the period division was the horizontal displacement of BR30. The parameters of a rectangular fault in each period were estimated from the three components of displacement.

**Figure 3:**
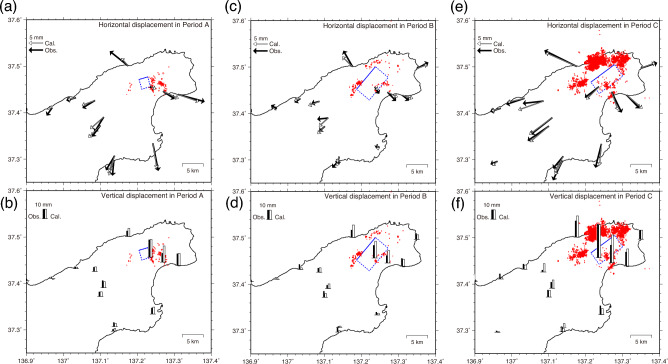
Estimated deformation sources and displacement for three periods. Open and solid symbols indicate observed and calculated displacement, respectively. Blue dotted rectangles represent tensile or shear tensile faults. Red dots indicate relocated epicenters of M ≥ 1.2 earthquakes. (**a**) Horizontal displacement for Period A (November 1–10, 2020, to March 1–10, 2021). (**b**) Vertical displacement for Period A. (**c**) Same as (**a**) but for Period B (March 1–10, 2021, to June 21–30, 2021). (**d**) Same as (**b**) but for Period B. (**e**) Same as (**a**) but for Period C (June 21–30, 2021, to June 9–18, 2022). (**f**) Same as (**b**) but for Period C.

Period A lasted from November 1–10, 2020, to March 1–10, 2021. Most earthquakes in this period occurred only in Cluster S. An uplift of ~ 20 mm was observed at BR30; however, significant horizontal displacement was not observed (Figs. 2a, 3a). We tested a shallow-dipping tensile crack and a southeast-dipping reverse fault as deformation sources and found that the former model better fit the observed displacement. The estimated crack was located in an aseismic region immediately west of Cluster S at a depth of ~ 16 km. The volumetric increase was ~ 1.4 × 10^7^ m^3^. Period B lasted from March 1–10, 2021, to June 21–30, 2021. The seismicity rate gradually increased, and earthquakes were activated in the order of Cluster W to Cluster N (Fig. 2a and Supplementary Fig. S2). A small southeastward displacement at BR30 (Figs. 2a, 3c) suggests a northeast source of BR30. The displacement was well reproduced by a southeast-dipping shear tensile fault with a reverse slip of 0.43 m and opening of 0.08 m (Fig. 3c,d), and it was located in the aseismic region among Clusters S, W, and N. The estimated moment magnitude was 5.70 assuming a rigidity of 30 GPa. Period C was from June 21–30, 2021, to June 9–18, 2022. The intensive seismicity expanded to Clusters N and NE. BR30 moved 6 mm southwest. The preferred model was a southeast-dipping shear tensile fault extending from Cluster S to Clusters N and NE with a reverse and left-lateral slip of 0.84 m and opening of 0.44 m. The estimated moment magnitude was 5.81. The opening component of the shear-tensile fault was minor and moderate in Periods B and C, respectively, and it improved data fitting, particularly in Period C. We performed a comparison of the data fitting for three different types of sources (e.g., point inflation, shear, and shear-tensile/tensile sources) using Akaike’s Information Criterion (AIC)^29^ (Supplementary Table S2). The difference in AIC is less than 2 during Period B, indicating all three sources can explain the GNSS displacement almost equally. However, point inflation and tensile sources representing volumetric changes reproduce the observed displacement significantly better than the shear source during Period A. The shear-tensile source significantly outperforms the other sources in explaining the displacement during Period C.

## Seismic swarm sequence scenario

**Figure 4:**
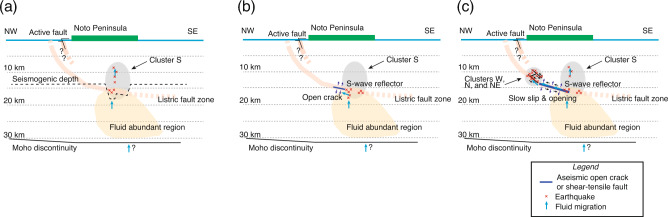
Schematic diagram of proposed mechanism of the Noto Peninsula earthquake swarm and transient deformation. (**a**) Pre-swarm stage from mid-2018 to the onset of the intense swarm. (**b**) Initial stage corresponding to Period A. (**c**) Main stage corresponding to Periods B and C.

We propose the following hypothetical scenario for the earthquake swarm based on the preferred model of deformation: in the swarm area, we infer that a southeast-dipping reverse fault zone extends from offshore of the northern coast of the peninsula to the upper and middle crust, based on the regional geology^9^ and hypocenter distribution (Supplementary Fig. S3). Crustal fluid is rich in the lower crust beneath Cluster S, as implied by the low seismic velocity^30,31^ and low electrical resistivity^32^. The latter study shows a conductive region with less than 20 m$\Omega$ distributes in and southwest of Cluster S below a depth of 15 km. A small volume of fluid was upwelled from the lower crust in the summer of 2018 and caused minor earthquake activity in Cluster S, which could not be detected by surface geodetic measurements (Fig. 4a). On November 30, 2020, a large volume of fluid was upwelled, which was accompanied by burst-type deep earthquakes of Cluster S. Overpressurized fluid ruptured an impermeable seal at a depth of ~ 16 km, and it spread in the permeable shallow-dipping fault zone in Period A (Figs. 3a,b, 4b). The fluid diffused in the fault zone and weakened the fault strength during Periods B and C, causing a submeter aseismic slip below the seismogenic depth (i.e., ~ 14 km) that relaxed the pre-swarm regional stress^23,24^ (Fig. 4c). The aseismic slip induced an increase in the surrounding stress and triggered the intense earthquake activities at the updip. The observed diffusive migration of earthquakes^25,26^ can be explained by both the expansion of the fluid-induced aseismic slip area^28^ and the weakening of the fault due to an increase in pore fluid pressure^33^. The fluid-induced aseismic slip occurred in all directions from the initial fluid-filled crack in Period B (Fig. 3c,d) and then eastward in Period C (Fig. 3e,f). The order of seismic activation from Cluster W to Cluster NE likely depends on the distance from the initial shallow-dipping crack northwest of Cluster S in Period A.

**Figure 5:**
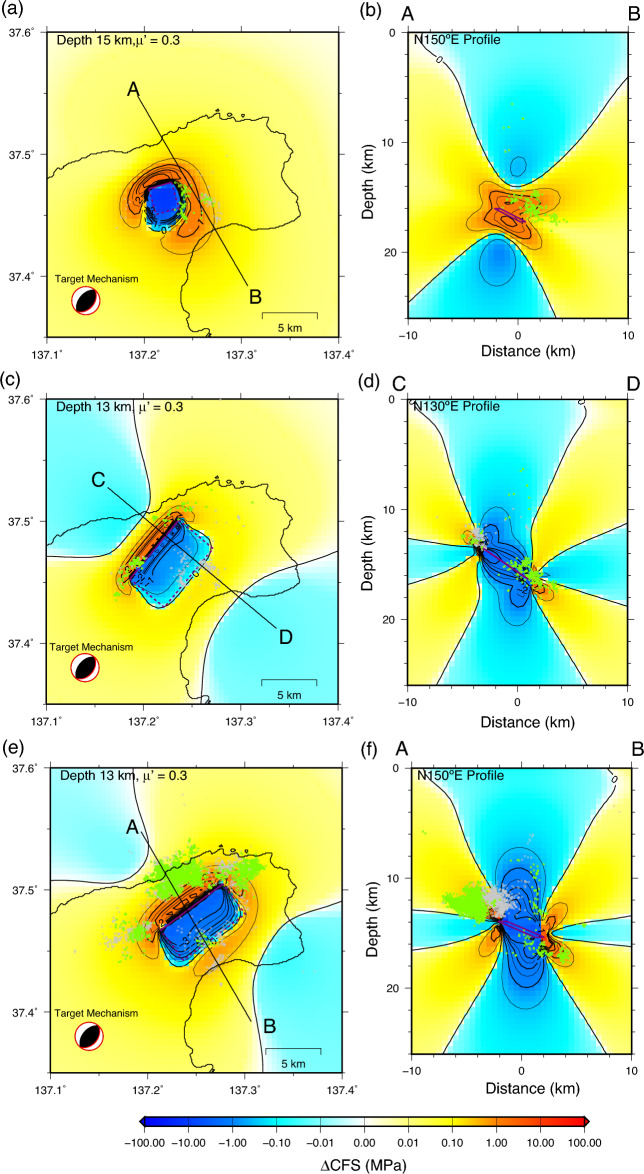
Coulomb stress changes (ΔCFS) due to estimated deformation sources and relocated hypocenters. (**a**) Horizontal profile at a depth of 15 km in Period A. Green and gray dots indicate the epicenters at depths within and beyond 1 km from the depth, respectively. (**b**) Vertical cross-section along the orientation N150° E (line A–B in (**a**)) in Period A. Green and gray dots indicate the hypocenter within and beyond 3 km from line A–B, respectively. (**c**) Same as (**a**) but at a depth of 13 km in Period B. (**d**) Same as (**b**) but along the orientation of N130° E (line C–D in (**a**)) in Period B. (**e**) Same as (**a**) but for Period C. (**f**) Same as (**b**) but for Period C.

A notable characteristic of the estimated deformation sources is that they are mostly located in an aseismic region surrounded by four earthquake clusters. This feature is robust if we assume different types of sources, including spherical inflation, pure tensile, and pure shear-slip sources. The calculated Coulomb failure stress change (ΔCFS) suggests stress increases and decreases in the deep and shallow parts of Cluster S, respectively (Fig. 5a,b), which explains the observed activation and quiescence below and above a depth of 14 km in Cluster S (Supplementary Fig. S1), respectively. The total moment of aseismic slip in Periods B and C was 1.10 × 10^18^ N m, which was five times the total seismic moment of the earthquakes (2.05 × 10^17^ N m: M_w_5.47) in the same period, converted using an empirical relation between the JMA magnitude and seismic moment^34^. A comparison between the seismic moment and the geodetically estimated moment for past slow slip events (SSEs) suggests that the aseismic moment often exceeds the seismic moment^35^. The moment ratio for the Noto swarm is in the range of the moment ratios for the past SSEs (i.e., 0.5–10^4^). The gradual increase in earthquakes in Clusters W, N, and NE was likely triggered by the stress increase due to the downdip slow aseismic slip. ΔCFS suggests that the stress increases up to a few MPa at the bottom of Clusters W, N, and NE in Periods B and C (Fig. 5c–f). However, we do not rule out the possibility that the earthquakes are directly induced by the fluid-induced aseismic slip and/or pore pressure increase in the region of Clusters W, N, and NE. Because our geodetic model assumes a simple uniform slip on a rectangular fault, small slip cannot be resolved in our analysis. Analysis to resolve a detailed aseismic slip distribution, including studies using small repeating earthquakes^36^ and high-precision tilt/strain measurements^37^, may provide further insight into the mechanism of earthquake triggering.

A number of examples of crustal seismic swarms related to upwelling fluids have been observed in non-volcanic areas, including Matsushiro (Japan)^38^ and L’aquila (Italy)^39^. Several earthquake swarms after the 2011 M_w_9.0 Tohoku-oki earthquake in northeastern Japan were likely triggered by an increase in pore fluid pressure^40,41^. However, transient deformation associated with seismic swarms is not usually detected geodetically except in the vicinity of volcanoes and major faults. The Noto swarm is characterized by a large volumetric increase (total 2.92 × 10^7^ m^3^ in Periods A–C), which is comparable to the seismic swarms in Matsushiro^38^ and volcanic areas^42^. The fluid causing the Noto swarm upwelled from the fluid-rich region in the lower crust and likely originated from the deep dehydration of the subducting slabs of the Pacific and/or Philippine Sea plates through mantle, as suggested by geochemical studies of the helium isotope around the swarm area^43^ and the Pb–Sr–Nd isotope in central Japan^44^, and a study of regional seismic tomography^45^. The location of the estimated open crack in Period A coincided with the S-wave reflector induced from the reflected phases in the seismograms^25^. These results support our scenario that the upwelling fluid pooled from the vicinity of Cluster S at the end of November 2020 and subsequently diffused and triggered an aseismic slip in the existing fault zone.

Although we modeled the observed deformation using a single shear tensile source in each period, the deformation likely represents a macroscopic view of numerous small shear tensile cracks in the fault zone. It is unrealistic to assume a single ten-square-kilometer fault accommodating both opening and shear slip, as a fault with overpressurized pore fluid pressure would slip rapidly to release shear stress. The characteristics of aseismic slip in the Noto Peninsula are not entirely typical for a SSE. For example, the duration (e.g., ≥ 16 months) relative to a moment is much longer than that predicted from the empirical scaling law of slow earthquakes^46^. Furthermore, the stress drop (e.g., ~ 5 MPa for Period C) was greater than that of a typical SSE and comparable to that of a regular earthquake^47^. These characteristics may result from a large and long supply of upwelling fluid.

## Seismic hazard implications

Numerous instances of seismic swarms preceding large and destructive earthquakes have been reported in the world^39,48,49^. Past earthquakes and active faults (Fig. 1) indicate the potential of an M ~ 7 earthquake around the swarm area. The rate of overall transient deformation decelerated after the M5.4 earthquake on June 19, 2022 (Fig. 2, Supplementary Fig. S5b). This implies that fluid supply from the lower crust has ceased by mid-2022, which is supported by the decay of deep intermittent earthquakes in Cluster S (Supplementary Fig. S2). Although the seismicity rate is still high as of February 2023, the overall seismicity rate can be expected to decrease unless additional fluid was supplied. However, the aseismic slip and opening in the fault zones have already stressed the surrounding faults at the seismogenic depth (Fig. 5) and advanced future fault rupture over time^50^. In addition, frequent dynamic stress perturbations due to strong seismic waves can also advance the time of fault rupture. It is difficult to quantitatively assess these “clock advance” effects for both static and dynamic changes because they depend not only on the stress amplitude but also on the onset time during the earthquake cycle^50^. Further analysis and monitoring of deformations and earthquakes will improve the assessment of seismic hazards.

## Deformation monitoring using a private-sector GNSS network

cGNSS networks have been constructed for public and academic purposes worldwide and have recently been developed for commercial purposes. The SoftBank GNSS network was essential in identifying transient deformation and inferring the source model in this study. For example, the uncertainties for the parameters estimated with the SoftBank stations are 2–4 times smaller than those without them if we assume a point inflation source for Period C. Although seasonal fluctuations are large at some SoftBank stations, partly attributed to differences in pre-processing among different GNSS networks, the repeatability of daily coordinates of SoftBank stations is comparable to that of GEONET and university stations (Supplementary Fig. S4). The root mean square errors (RMSEs) averaged for the 14 SoftBank stations after removing a linear component for 2021 were 1.9, 2.0, and 5.3 mm for the north, east, and vertical components, respectively, while the values for the 10 GEONET stations were 1.7, 1.7, and 5.5 mm, respectively. Although the quality check for each station and/or component is necessary, this study demonstrates the capability of Softbank GNSS stations to monitor crustal deformation over a timescale of years. The utilization of GNSS networks operated by the private sector can dramatically improve the spatial resolving power of deformation monitoring and deepen our understanding of not only shallow tectonic processes but also of hydrological response, atmospheric phenomena, and ionospheric phenomena.

## Methods

### Hypocenter relocation

We attempted to relocate 7560 events with magnitudes ≥ 1.2 that occurred between January 2018 and November 2022 in or around the northeastern tip of the Noto Peninsula using the double–differential method (hypoDD)^51^. The hypocenter and arrival time data of the stations in or around the peninsula (Supplementary Fig. S8a), reported by the JMA, were used as the catalog data. The velocity structure provided by the JMA was referenced^52^ (Supplementary Fig. S8b). The number of differential travel times of the catalog data was 493,494 and 486,132 for the P- and S-waves, respectively.

We also calculated precise travel time differences of the P- and S-waves using velocity waveforms in the 1–8 Hz frequency bands^11,53,54^ for pairs of events. We utilized the differential travel times for an event pair in hypoDD calculation if there were over 12 phase pairs presented with an average squared coherency ≥ 0.8 in the 1–8 Hz frequency bands. Finally, we obtained 5,041,816 and 3,904,866 differential travel times for P- and S-waves from the waveform analysis, respectively. Consequently, we precisely relocated 6705 events. The root mean square of the travel time residuals decreased from 0.1362 to 0.1002 s for the catalog data and from 0.1785 to 0.0238 s for the waveform analysis data.

### GNSS data processing

The daily coordinates of 30 GNSS stations (Supplementary Fig. S9) were estimated using precise point positioning with ambiguity resolution implemented in the GipsyX Ver. 1.4 software package^55^. We used the VMF1 model for tropospheric correction and applied higher-order ionosphere correction. To correct ocean tide loading, we used FES2014b^56^ by applying parameters downloaded from the Onsala Space Observatory (http://holt.oso.chalmers.se/loading/). The daily coordinates were transformed into the IGS14 reference frame using the Helmert transformation parameters provided by the Jet Propulsion Laboratory. To extract the transient deformation associated with the earthquake swarm, we removed secular, seasonal, and long-term postseismic deformation associated with the 2011 M_w_9.0 Tohoku-oki earthquake before November 2020. For the GEONET sites, we fitted linear, logarithmic, exponential^57^, and sinusoidal functions to the daily coordinates from March 12, 2011, to October 31, 2020, and removed them from the observed coordinates to extract the transient deformation. However, we could not estimate the pre-swarm deformation from data collected at SoftBank and university sites because the data did not cover a sufficiently long time period. Therefore, we removed only the linear component by interpolating the velocities at the surrounding GEONET sites from November 2017 to October 2020 using basis function expansion^58^. Finally, we calculated common-mode noise by stacking daily coordinates at five stable sites located 50–100 km away from the swarm area and applied spatial filtering^59^ to remove common-mode noise from all stations.

Stations BR16 and SZOT are located northwest of the earthquake swarm area (Supplementary Fig. S10). Despite being approximately 165 m apart, their baseline changes show a relative displacement of ≥ 10 mm from September 2021 to December 2022 (Supplementary Fig. S10b). This displacement cannot be explained by a subsurface tectonic source. We suspect that monument instability is likely the cause of the anomaly observed at BR16, although the data collected at BR16 are valuable because they record the transient deformation from the beginning of November 2020 (Fig. 2a). This information is important considering that SZOT was installed in September 2021. Therefore, we applied a site-specific correction to the daily coordinates of BR16. We assumed that BR16 and SZOT recorded the same deformation and local non-tectonic displacement of BR16 annually. The relative displacement between BR16 and SZOT was extrapolated from a 14-day average median spanning from November 1, 2021, to October 31, 2022 (Supplementary Fig. S10b). Then, we subtracted the relative displacement from the preprocessed BR16 coordinates (Supplementary Fig. S10a) and derived the corrected BR16 coordinates (Supplementary Fig. S10c). The corrected coordinates were used for further analysis.

### Fault model inversion

We assumed a rectangular dislocation source in a uniform elastic half-space^60^ and estimated its parameters by incorporating prior information on the parameters using the nonlinear inversion method of Matsu’ura and Hasegawa^61^. The rectangular fault has a uniform slip and/or open component. The displacements and their uncertainties are calculated from differences between the averages of the daily coordinates and their standard deviation in two periods. The displacements are inversely weighted by their uncertainties in the inversion. We used the hypocenter distribution and regional tectonic settings as prior information in the inversion (Supplementary Table S3). The estimated parameters for each period are listed in Table 1. We calculated approximate stress drop ($\Delta \sigma$) by using $\Delta \sigma \approx \mu D/L$, where $\mu$ is the shear modulus, $D$ is the slip and $L$ is the square root of the slip area. The stress drop derived from the estimated parameters for Periods B and C are ~ 2 MPa and ~ 5 MPa, respectively.

**Table 1:** Estimated parameters and one-standard deviation uncertainties for the deformation sources in three periods.

	Longitude (°)	Latitude (°)	Depth (km)	Length (km)	Width (km)	Strike (º)	Dip (º)	Rake (º)	Slip (m)	M_w_^a^	Open (m)	ΔV (×10^7^ m^3^)
Period A	137.205 ± 0.023	37.471 ± 0.023	16.0 ± 2.2	2.2 ± 4.8	2.7 ± 4.7	74 ± 14	26 ± 6				2.55 ± 7.15	1.51
Period B	137.186 ± 0.019	37.452 ± 0.026	13.5 ± 1.8	7.0 ± 4.6	4.9 ± 4.3	40 ± 9	35 ± 7	93 ± 9	0.44 ± 0.49	5.70	0.08 ± 0.10	0.27
Period C	137.214 ± 0.019	37.467 ± 0.021	13.9 ± 1.4	7.0 ± 3.7	3.7 ± 4.2	53 ± 9	23 ± 7	126 ± 11	0.84 ± 1.18	5.81	0.44 ± 0.59	1.14

^a^Rigidity is assumed to be 30 GPa.

### Calculation of Coulomb stress change (ΔCFS)

Coulomb failure stress change (CFS) ^62^ is defined as

1 $${\Delta }CFS = {\Delta }\tau_{s} + \mu^{\prime}\Delta \sigma_{n}$$

where $\Delta {\tau }_{s}$, ${\Delta \sigma }_{n}$, and μ′ represent changes in the shear stress and normal stress (positive in extension) for the target fault geometry and apparent friction coefficient, respectively. We calculated stress change in a uniform elastic half-space^63^. The fault geometry of the receiver fault was fixed at 40°, 40°, and 90° for strike, dip, and rake, respectively, which is concordant with the focal mechanism around the swarm area (Fig. 1). The apparent friction coefficient was assumed to be 0.3.

### Addendum

On May 5, 2023, during the revision of this paper, an M6.5 (M_w_6,2) earthquake occurred in the Noto Peninsula. This earthquake resulted in 38 casualties and damage to more than six hundred buildings. Its aftershock area overlapped Clusters N and NE and further expanded to a northern offshore region (See the evaluation by the Earthquake Research Committee, https://www.static.jishin.go.jp/resource/monthly/2023/2023_ishikawa_1.pdf). Further analysis of the transient deformation and seismicity associated with the Noto swarm is essential to resolve the mechanism and possible triggering processes of the earthquake.

## Supplementary Information

Supplementary Information.

## Data Availability

GNSS data from the SoftBank observation network are not publicly available because they are provided by SoftBank Corp. and ALES Corp. to the “Consortium to utilize the SoftBank original reference sites for Earth and Space Science” under the associated contract. However, they can be made available from the corresponding author upon reasonable request and with the permission of SoftBank Corp. and ALES Corp. GNSS data from university stations and the earthquake catalog relocated in this study are available from the corresponding author. GNSS data from GSI stations, earthquake catalog data, arrival times reported by JMA, focal mechanisms determined by NIED and GCMT, and seismic waveform data can be downloaded from the corresponding websites: (https://terras.gsi.go.jp/, https://www.data.jma.go.jp/eqev/data/bulletin/index_e.html, https://www.fnet.bosai.go.jp/event/search.php?LANG=en, https://www.globalcmt.org/, https://hinetwww11.bosai.go.jp/auth/?LANG=en).
