# Supplementary material for: Episodic transient deformation revealed by the analysis of multiple GNSS networks in the Noto Peninsula, central Japan
Source: Sci Rep. 2023 Jun 12;13:8381. doi: 10.1038/s41598-023-35459-z (PMC10260969; doi:10.1038/s41598-023-35459-z)
Supplement: Supplementary file 1 — Supplementary Information. [file 41598_2023_35459_MOESM1_ESM.pdf]

Episodic transient deformation revealed by the analysis of multiple GNSS networks in the Noto Peninsula, central Japan

Takuya Nishimura*[1], Yoshihiro Hiramatsu[2], and Yusaku Ohta[3]

[1]Disaster Prevention Research Institute, Kyoto University, Gokasho, Uji, Kyoto Prefecture 611-0011, Japan

[2]Institute of Science and Engineering, Kanazawa University, Kanazawa, Kakuma-cho, Ishikawa Prefecture 920-1192, Japan

[3]Graduate School of Science, Tohoku University, Aza-Aoba 6-6, Aramaki, Aoba-ku, Sendai, Miyagi Prefecture 980-8578, Japan

*Corresponding author (nishimura.takuya.4s@kyoto-u.ac.jp)

Contents:

Figure S1: Temporal depth sequence of earthquakes in Cluster S.

Figure S2: The weekly number of M≥1 earthquakes in Clusters NE, N, W, and S (Fig. 1b).

Figure S3: Vertical cross-section of relocated hypocenters in N60ºE direction.

Figure S4: Observed and pre-processed time-series of GNSS daily positions.

Figure S4: Distribution of GNSS stations used in this study.

Figure S5: Displacement during and after the M5.4 earthquake on June 19, 2022.

Figure S6: Displacement predicted by a point inflation source, opening of a horizontal crack, and a reverse fault slip.

Figure S7: Close-up view of horizontal displacement predicted by a point inflation source, opening of a horizontal crack, and a reverse fault slip.

Figure S8: Seismic station distribution and velocity structure used in this study.

Figure S9: Distribution of GNSS stations used in this study.

Figure S10: Time-series of BR16 before and after a site-specific correction.

Table S1 Parameters for deformation sources in Fig. S6.

Table S2 Parameters for different-type deformation sources in Periods A-C.

Table S3 Initial parameters and their prior constraints for deformation sources in three periods.

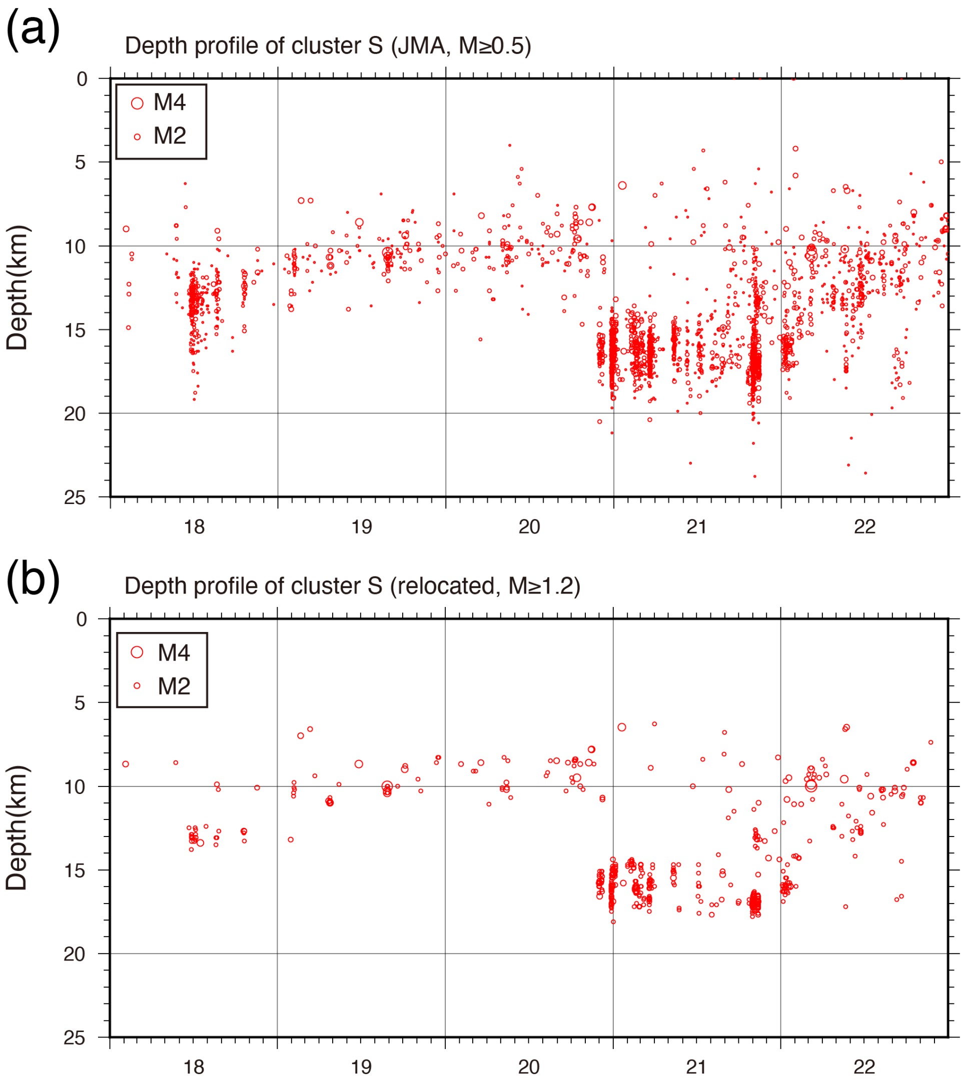

Fig. S1 Temporal depth sequence of earthquakes in Cluster S. (a) M≥0.5 earthquakes using the JMA catalog. (b) M≥1.2 earthquakes using the relocated catalog.

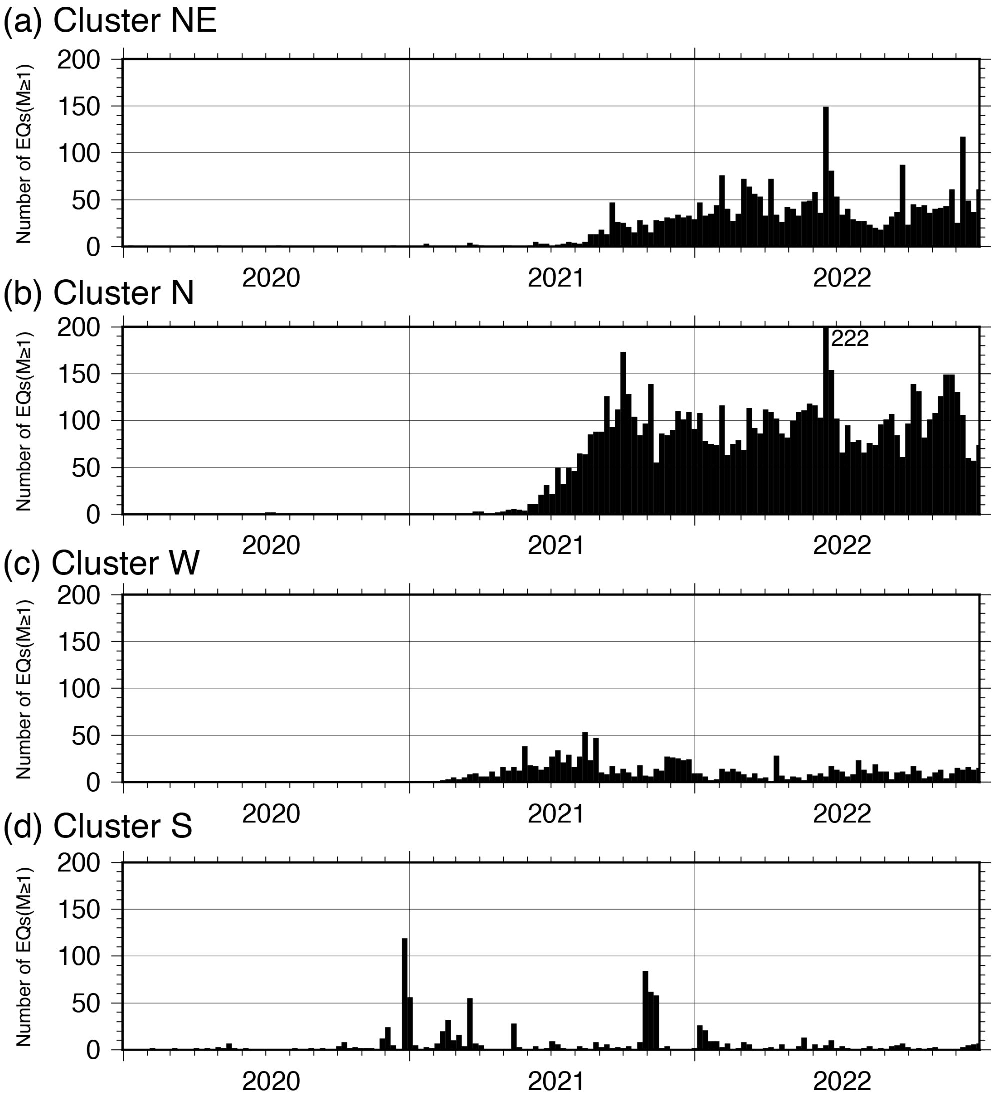

Fig. S2 The weekly number of M≥1 earthquakes in Clusters NE, N, W, and S (Fig. 1b).

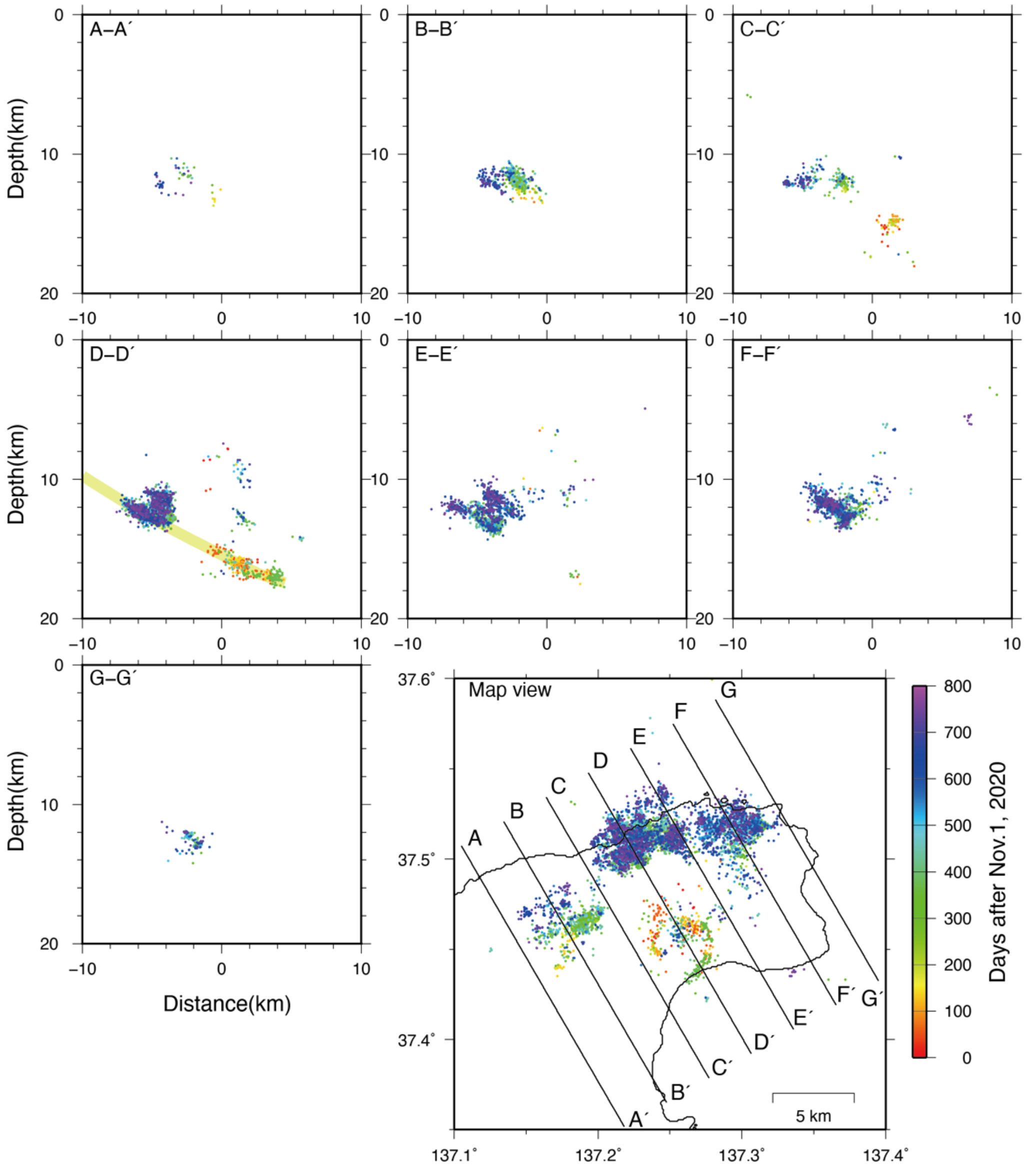

Fig. S3 Vertical cross-section of relocated hypocenters in N60ºE direction. The color represents days after November 1, 2020. A possible southeast-dipping fault zone is indicated in the cross-section D-D´.

00BR0L.neu

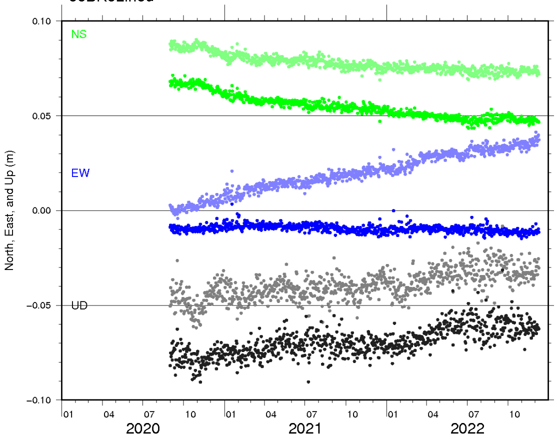

00BR0T.neu

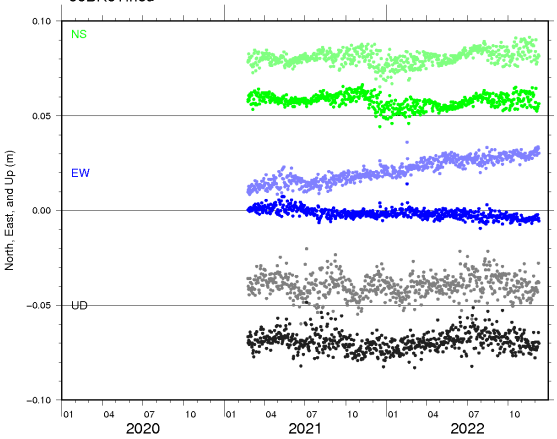

00BR0V.neu

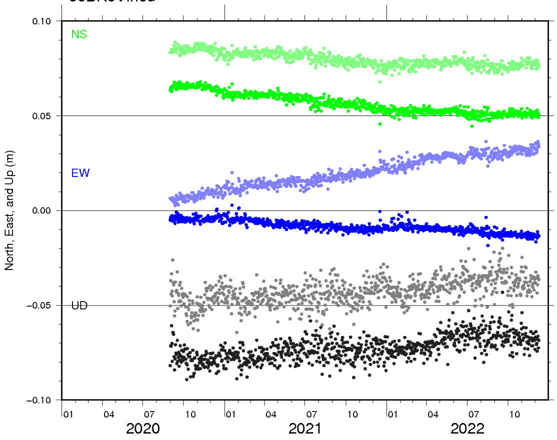

00BR0V.neu

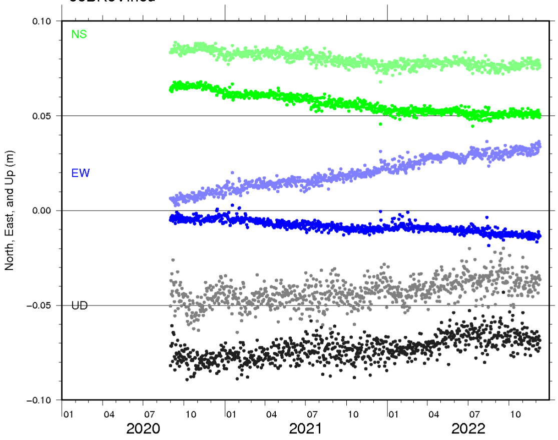

00BR1H.neu

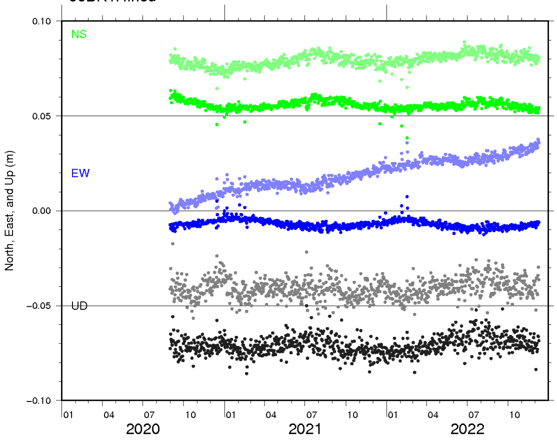

00BR1Q.neu

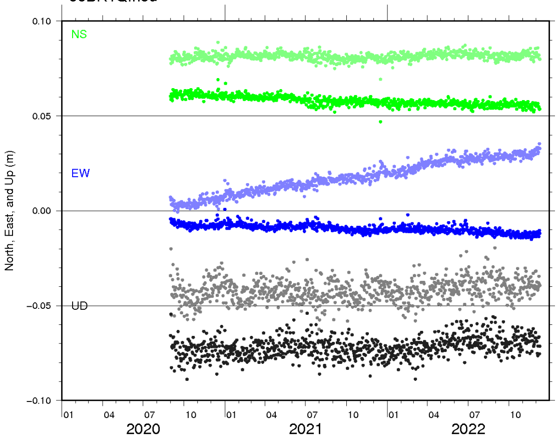

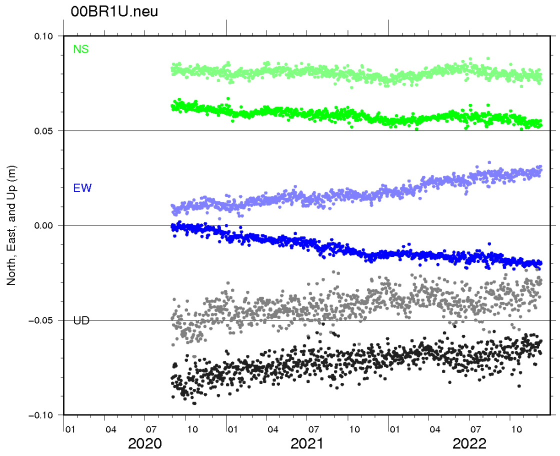
00BR1U.neu
NS
EW
UD
North, East, and Up (m)
0.10
0.05
0.00
−0.05
−0.10
01 04 07 10 01 04 07 10 01 04 07 10
2020
2021
2022

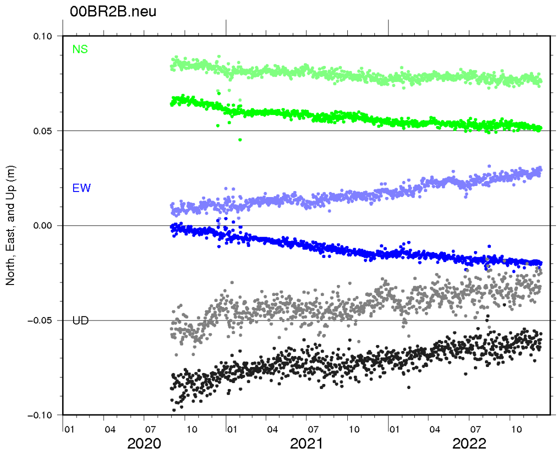
00BR2B.neu
NS
EW
UD
North, East, and Up (m)
0.10
0.05
0.00
−0.05
−0.10
01 04 07 10 01 04 07 10 01 04 07 10
2020
2021
2022

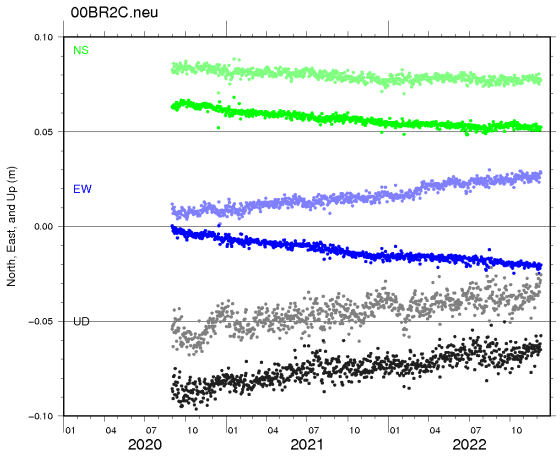
00BR2C.neu
NS
EW
UD
North, East, and Up (m)
0.10
0.05
0.00
−0.05
−0.10
01 04 07 10 01 04 07 10 01 04 07 10
2020
2021
2022

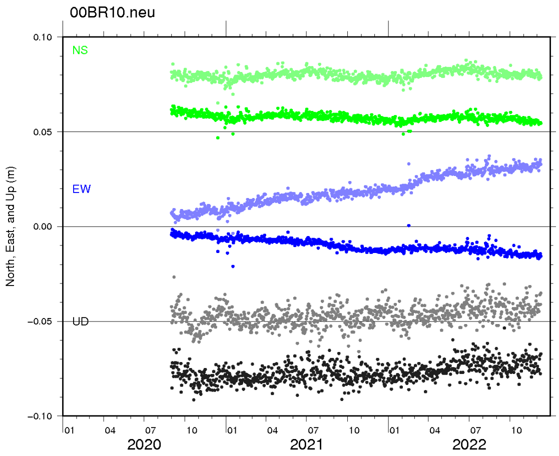
00BR10.neu
NS
EW
UD
North, East, and Up (m)
0.10
0.05
0.00
−0.05
−0.10
01 04 07 10 01 04 07 10 01 04 07 10
2020
2021
2022

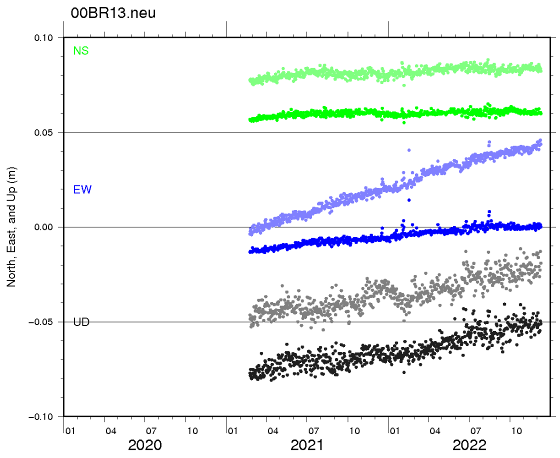
00BR13.neu
NS
EW
UD
North, East, and Up (m)
0.10
0.05
0.00
−0.05
−0.10
01 04 07 10 01 04 07 10 01 04 07 10
2020
2021
2022

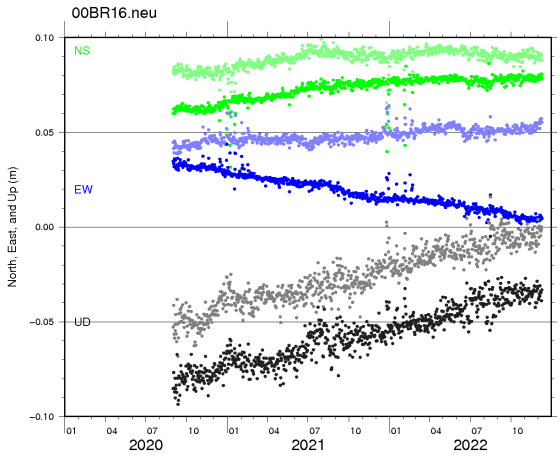
00BR16.neu
NS
EW
UD
North, East, and Up (m)
0.10
0.05
0.00
−0.05
−0.10
01 04 07 10 01 04 07 10 01 04 07 10
2020
2021
2022

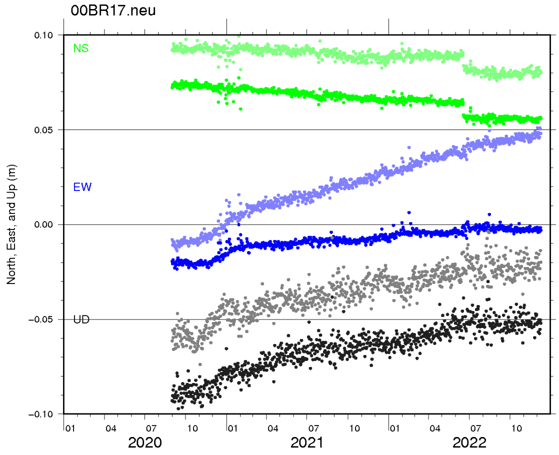
00BR17.neu
NS
EW
UD
North, East, and Up (m)
0.10
0.05
0.00
−0.05
−0.10
01 04 07 10 01 04 07 10 01 04 07 10
2020
2021
2022

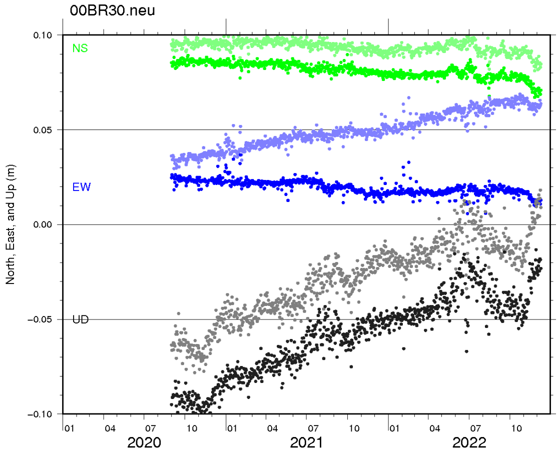
00BR30.neu
NS
EW
UD
North, East, and Up (m)
0.10
0.05
0.00
−0.05
−0.10
01 04 07 10 01 04 07 10 01 04 07 10
2020
2021
2022

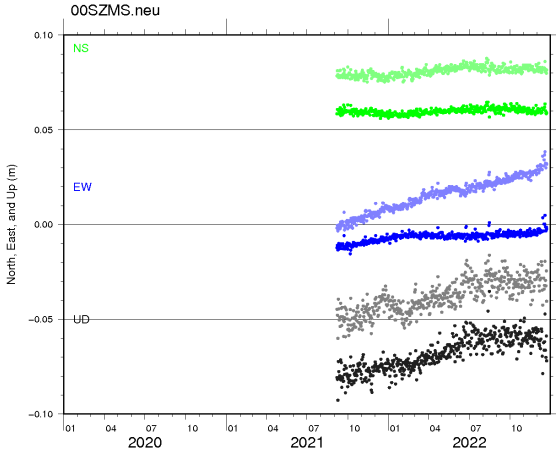
00SZMS.neu
NS
EW
UD
North, East, and Up (m)
0.10
0.05
0.00
−0.05
−0.10
01 04 07 10 01 04 07 10 01 04 07 10
2020
2021
2022

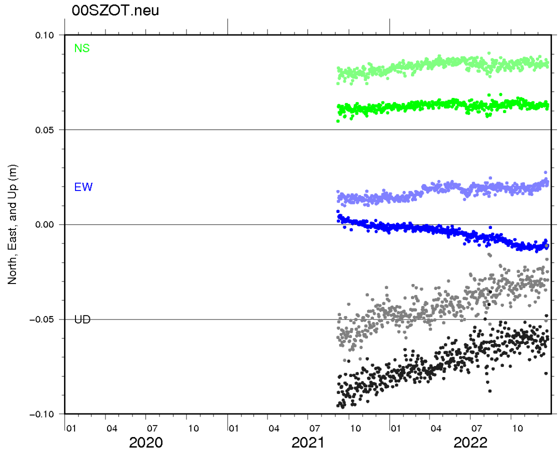
00SZOT.neu
NS
EW
UD
North, East, and Up (m)
0.10
0.05
0.00
−0.05
−0.10
01 04 07 10 01 04 07 10 01 04 07 10
2020
2021
2022

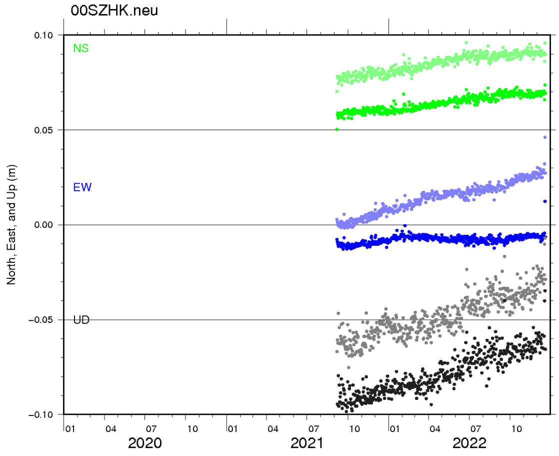
00SZHK.neu
NS
EW
UD
North, East, and Up (m)
0.10
0.05
0.00
−0.05
−0.10
01 04 07 10 01 04 07 10 01 04 07 10
2020
2021
2022

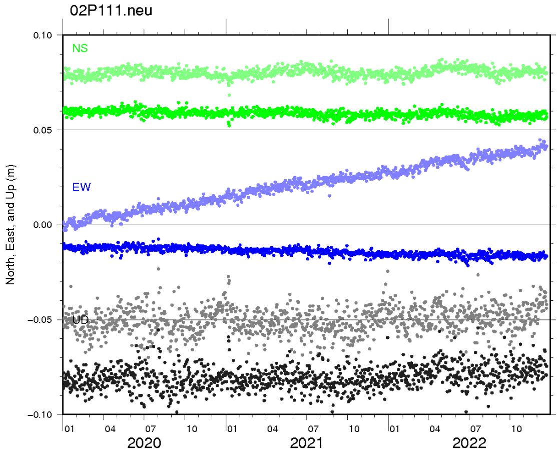
02P111.neu
NS
EW
UD
North, East, and Up (m)
0.10
0.05
0.00
−0.05
−0.10
01 04 07 10 01 04 07 10 01 04 07 10
2020
2021
2022

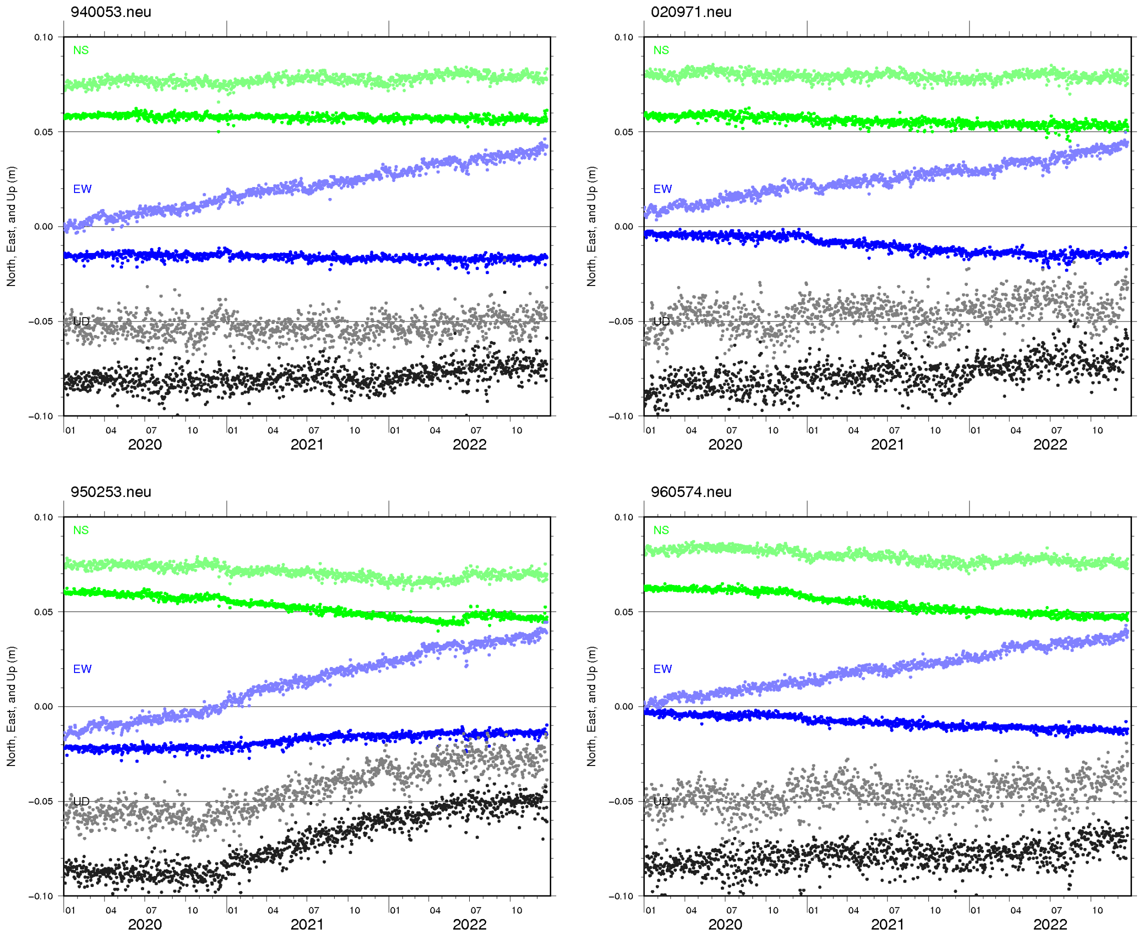

Fig. S4 Observed and pre-processed time-series of GNSS daily positions. Thin and thick dots show the original AM-referenced and pre-processed daily positions of each component.

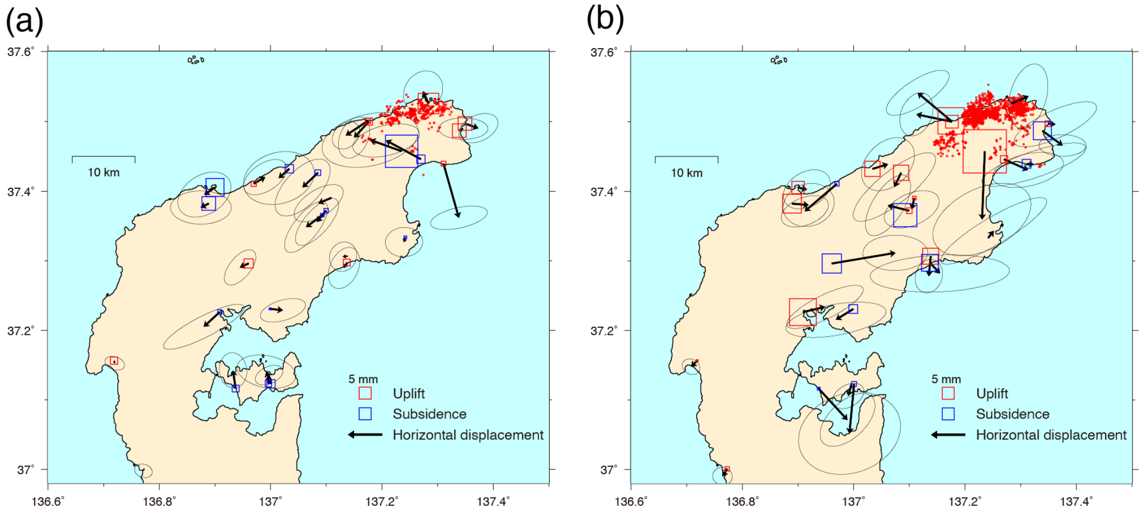

Fig. S5 Displacement during and after the M5.4 earthquake on June 19, 2022. (a) Displacement from June 9-18 to June 21-30, 2022. Red dots show relocated epicenters of M≥1.2 earthquakes from June 9 to June 30, 2022. (b) Displacement from June 21-30 to December 22-31, 2022. Red dots show relocated epicenters of M≥1.2 earthquakes from June 21 to November 30, 2022.

(a)

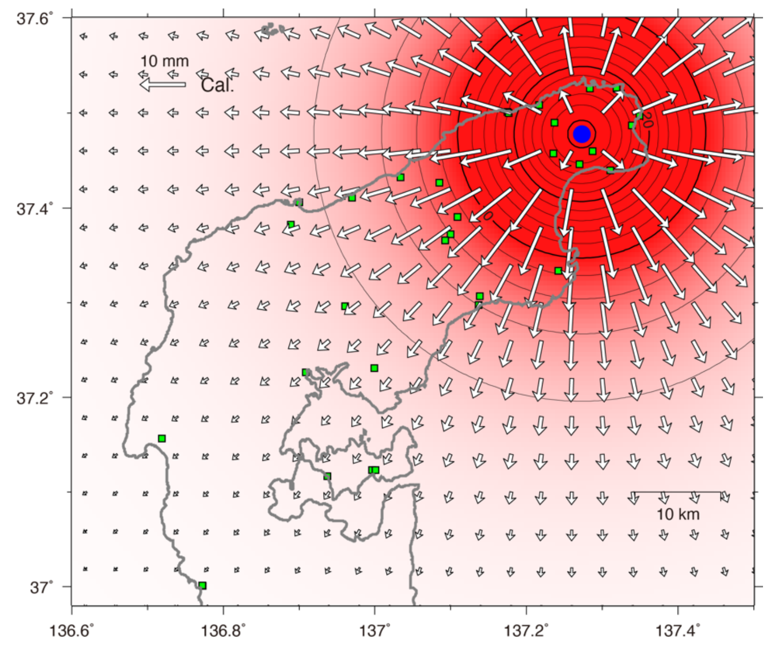
37.6°
37.4°
37.2°
37°
136.6°
136.8°
137°
137.2°
137.4°
10 mm
Cal.
10 km
20
10

(b)

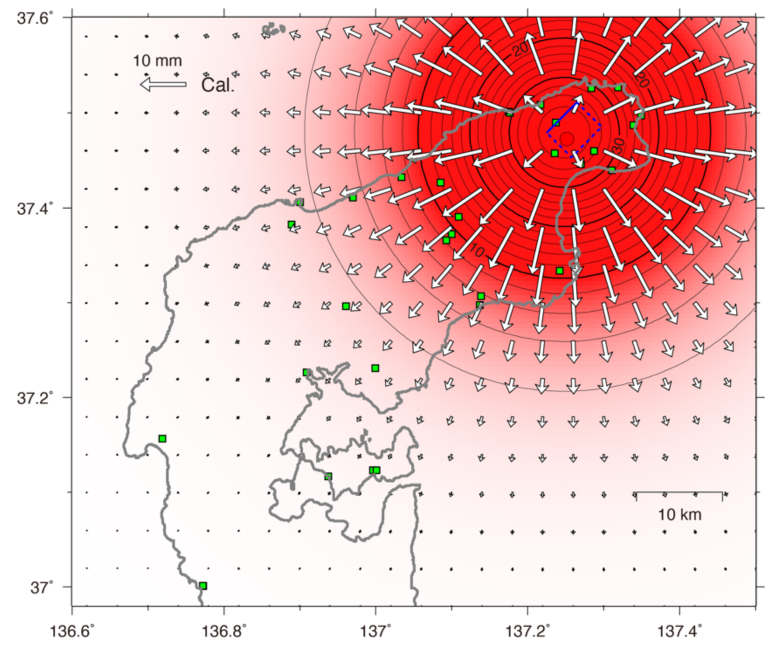
37.6°
37.4°
37.2°
37°
136.6°
136.8°
137°
137.2°
137.4°
10 mm
Cal.
10 km
20
30
10

(c)

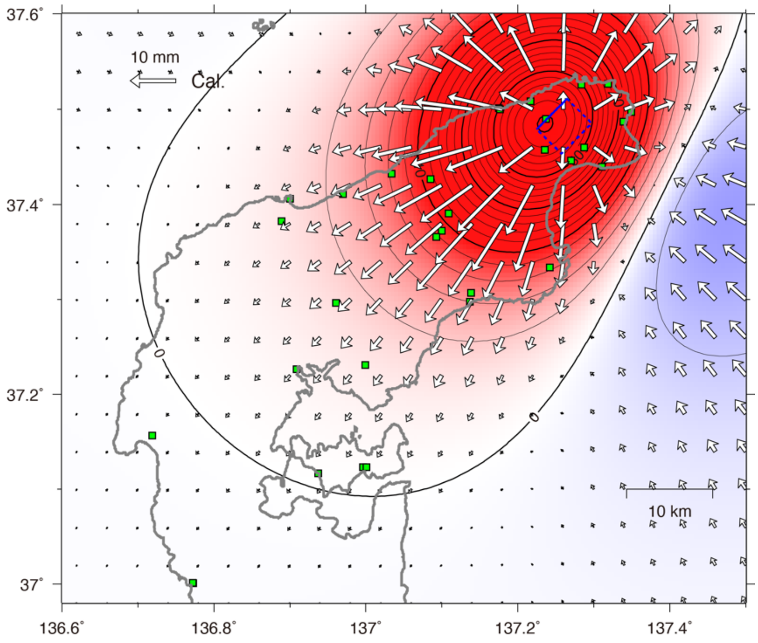

Fig. S6 Displacement predicted by a point inflation source (a), opening of a horizontal crack (b), and a reverse fault slip (c). These models roughly reproduce the displacement observed from February 2021 to February 2022. Red and blue regions represent uplift and subsidence, respectively. Green squares indicate GNSS stations. Fault parameters are shown in Table S1.

(a)

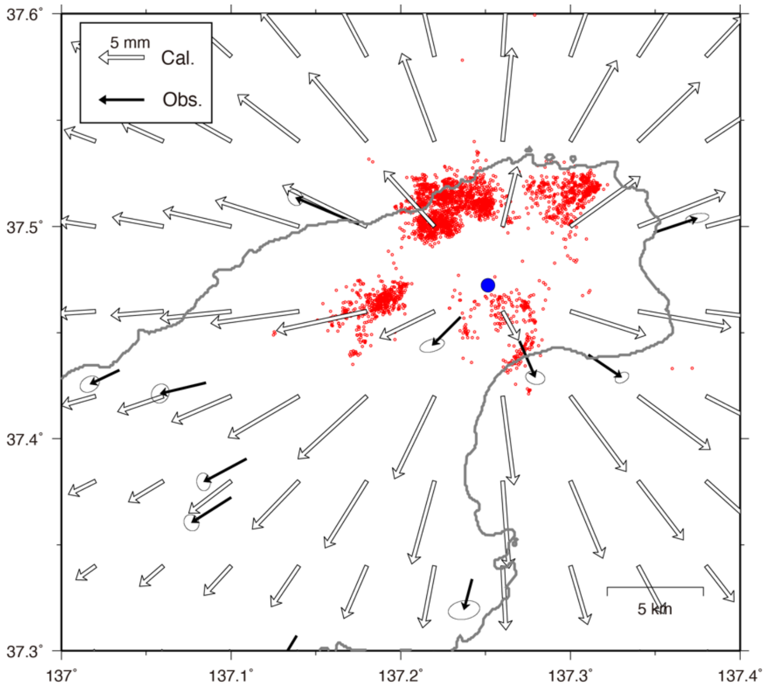
5 mm
Cal.
Obs.
5 km
37.6°
37.5°
37.4°
37.3°
137°
137.1°
137.2°
137.3°
137.4°

(b)

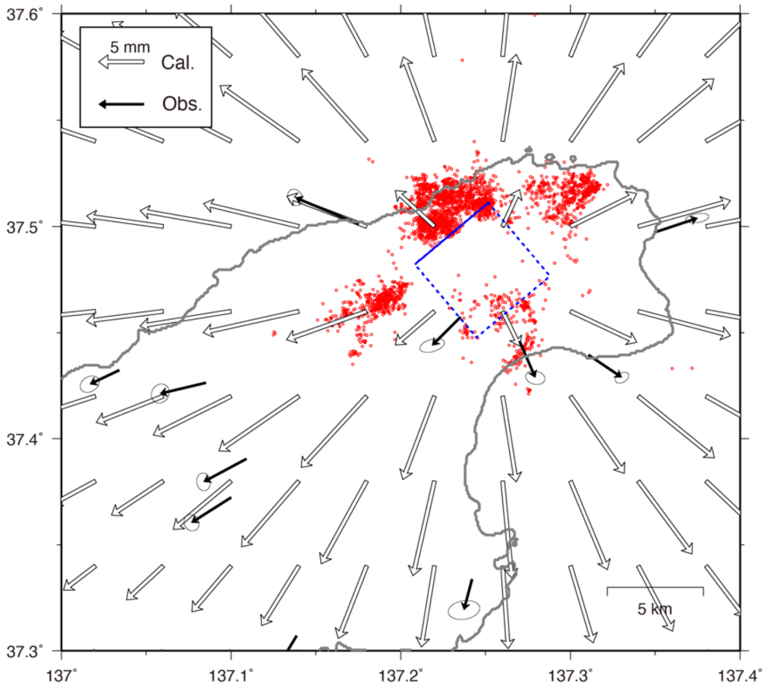
5 mm
Cal.
Obs.
5 km
37.6°
37.5°
37.4°
37.3°
137°
137.1°
137.2°
137.3°
137.4°

(c)

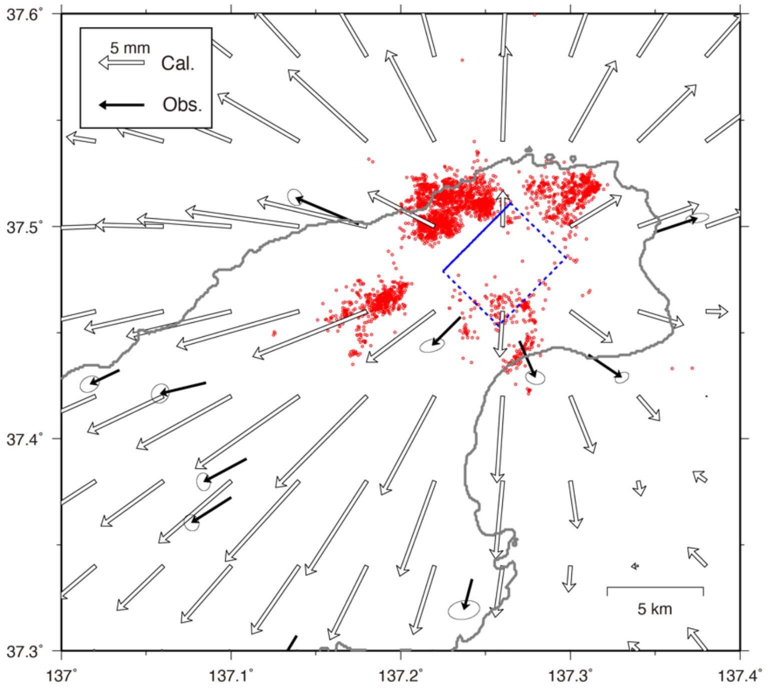

Fig. S7 Close-up view of horizontal displacement predicted by a point inflation source (a), opening of a horizontal crack (b), and a reverse fault slip (c). These models are the same as those shown in Fig S6. Fault parameters are shown in Table S1. Open and solid vectors represent predicted and observed displacements, respectively. Note that the locations of the source centroids are well constrained by the observed GNSS displacements because the predicted displacements show a radial pattern from the source centroid.

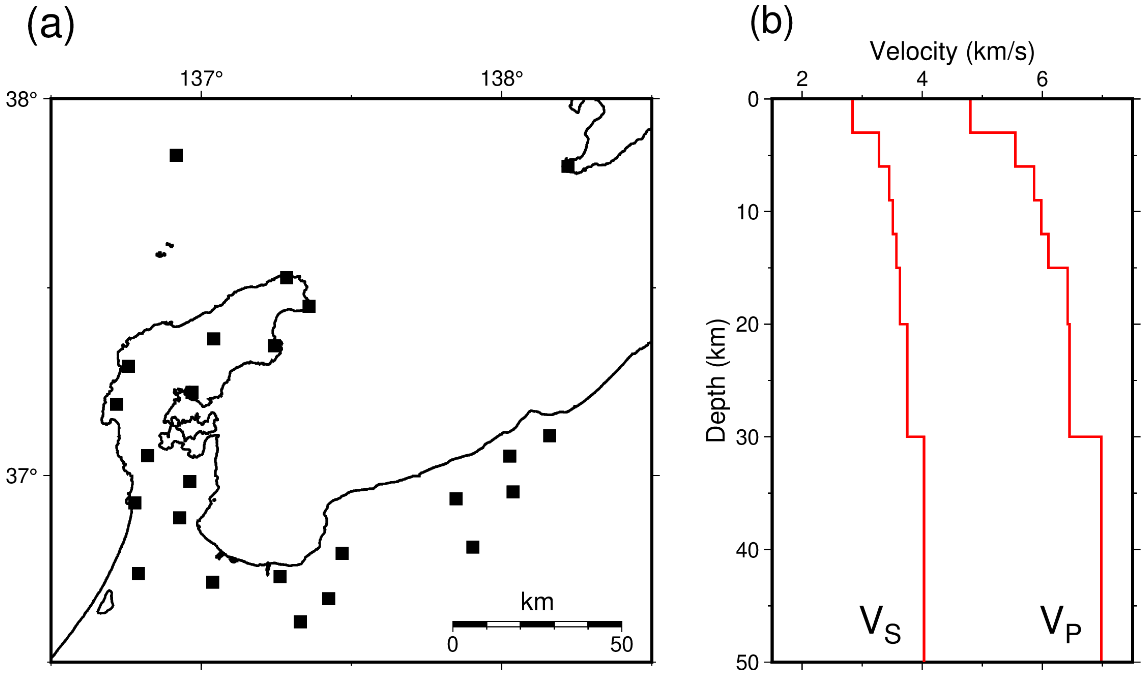

Fig. S8 Seismic station distribution (a) and velocity structure (b) used in this study.

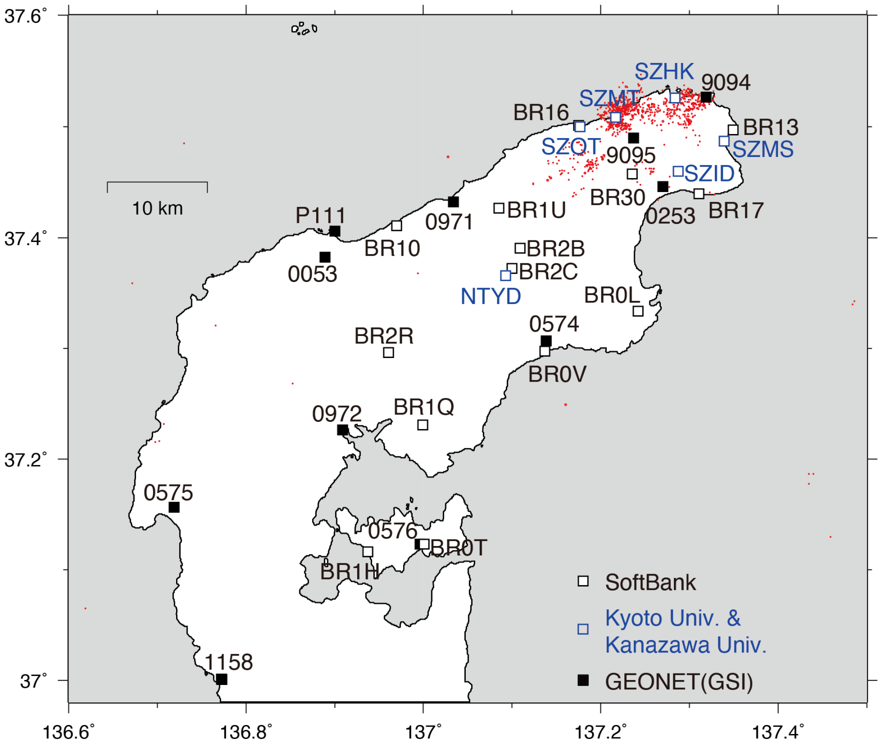

Fig. S9 Distribution of GNSS stations used in this study. Four-digit codes indicate GNSS station ID. Red dots show epicenters of M≥2 earthquakes with a depth of ≤20 km from November 2020 to December 2022.

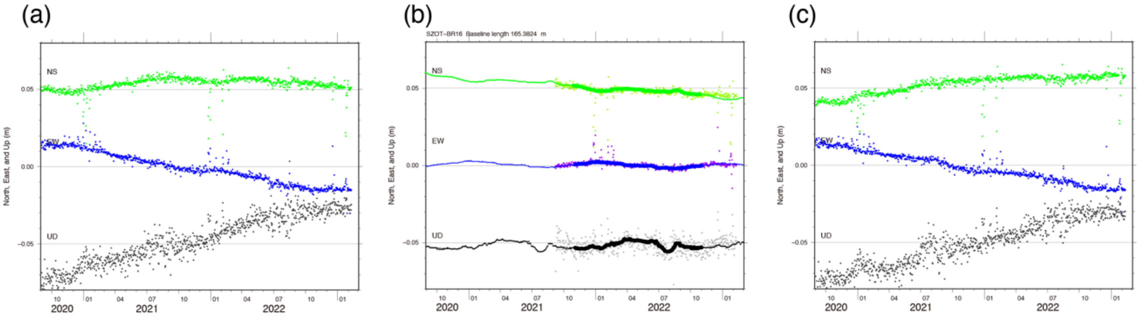

Fig. S10 Time-series of BR16 before and after a site-specific correction. (a) Pre-processed time-series of BR16 before the correction. (b) Time-series of relative coordinates between BR16 and SZOT. Dots are daily relative coordinates. Thick lines represent 14-day moving median of daily relative coordinates from November 1, 2021 to October 31, 2022. Thin lines represent presumed local site-specific movements of BR16. (c) Corrected time-series of BR16.

Table S1 Parameters for deformation sources in Fig. S6.

	Longitude (°)	Latitude (°)	Depth (km)	Length (km)	Width (km)	Strike (°)	Dip (°)	Rake (°)	Slip (m)	Open (m)	ΔV ($x10^7 m^3$)
Point inflation	137.273	37.478	13.8								2.42
Opening crack	137.208	37.482	20.0	5.0	5.0	50	0			1.30	3.25
Reverse fault	137.225	37.479	16.3	5.0	5.0	45	36	105	2.10		

Table S2 Parameters for different-type deformation sources in Periods A-C.

Period	Source type	Longitude (°)	Latitude (°)	Depth (km)	Length (km)	Width (km)	Strike (°)	Dip (°)	Rake (°)	Slip (m)	Open (m)*	AIC
A	Point inflation	137.237	37.459	11.8	-	-	-	-	-	-	1.20	198.58
	Shear	137.201	37.462	15.1	3.5	4.9	34	50	106	1.75	-	232.67
	Tensile	137.205	37.471	16.0	2.2	2.7	74	26	-	-	2.55	199.07
B	Point inflation	137.186	37.452	10.4	-	-	-	-	-	-	0.57	205.28
	Shear	137.192	37.454	14.0	6.3	4.6	39	39	91	0.65	-	206.03
	Shear-tensile	137.186	37.452	13.5	7.0	4.9	40	35	93	0.44	0.08	206.75
C	Point inflation	137.471	37.252	11.3	-	-	-	-	-	-	1.44	249.50
	Shear	137.228	37.459	15.1	6.3	2.4	43	35	110	2.69	-	230.14
	Shear-tensile	137.214	37.467	13.9	7.0	3.7	53	23	126	0.84	0.44	212.08

*Volume change (Unit: 10^7 m^3) for the point inflation source.

Table S3 Initial parameters and their prior constraints for deformation sources in three periods.

	Longitude (°)	Latitude (°)	Depth (km)	Length (km)	Width (km)	Strike (°)	Dip (°)	Rake (°)	Slip (m)	Open (m)
Period A	137.250 ±9.9	37.470 ±9.9	16.0 ±3.0	3.0 ±5.0	3.0 ±5.0	50 ±99	0 ±99			0.00 ±99.9
Period B	137.230 ±9.9	37.450 ±9.9	13.0 ±3.0	5.0 ±5.0	5.0 ±5.0	40 ±10	40 ±10	80 ±99	0.00 ±99.9	0.00 ±99.9
Period C	137.200 ±9.9	37.480 ±9.9	13.0 ±3.0	5.0 ±5.0	5.0 ±5.0	50 ±10	40 ±10	120 ±99	0.00 ±99.9	0.00 ±99.9
